# Parameter free AEWMA control chart for dispersion in semiconductor manufacturing

**DOI:** 10.1038/s41598-024-61408-5

**Published:** 2024-05-07

**Authors:** Abdullah A. Zaagan, Imad Khan, Ali Rashash R. Alzahrani, Bakhtiyar Ahmad

**Affiliations:** 1 Department of Mathematics, Faculty of Science, Jazan University, P.O. Box 2097, 45142 Jazan, Kingdom of Saudi Arabia; 2 Department of Statistics, Abdul Wali Khan University Mardan, Mardan, 23200 Pakistan; 3 Mathematics Department, Faculty of Sciences, Umm Al-Qura University, Makkah, Kingdom of Saudi Arabia; 4 Higher Education Department, Kabul, 1001 Afghanistan

**Keywords:** Statistical process control, Average run length, Control chart, AEWMA, EWMA, Process dispersion, Engineering, Mathematics and computing, Physics

## Abstract

The study presents a new parameter free adaptive exponentially weighted moving average (AEWMA) control chart tailored for monitoring process dispersion, utilizing an adaptive approach for determining the smoothing constant. This chart is crafted to adeptly detect shifts within anticipated ranges in process dispersion by dynamically computing the smoothing constant. To assess its effectiveness, the chart’s performance is measured through concise run-length profiles generated from Monte Carlo simulations. A notable aspect is the incorporation of an unbiased estimator in computing the smoothing constant through the suggested function, thereby improving the chart’s capability to identify different levels of increasing and decreasing shifts in process dispersion. The comparison with an established adaptive EWMA-S^2^ dispersion chart highlights the considerable efficiency of the proposed chart in addressing diverse magnitudes of process dispersion shifts. Additionally, the study includes an application to a real-life dataset, showcasing the practicality and user-friendly nature of the proposed chart in real-world situations.

## Introduction

Statistical process control (SPC) is a systematic quality management method with the aim of ensuring consistent, high-quality production. Developed by Shewhart and expanded by Deming, SPC involves the use of statistical techniques to monitor and analyze process variations. Key components include the use of control charts that visually represent process variability over time, with a centerline indicating the process mean and control limits delineating acceptable variation. SPC distinguishes between common and special cause variations, enabling continuous monitoring and early detection of deviations. If deviations occur, corrective actions are taken to maintain process control. Used in various industries, SPC contributes to improved product quality, error reduction and overall process optimization, promoting a culture of continuous improvement and customer satisfaction. Its cornerstone, quality control charts, has evolved from classical Shewhart charts, reflecting ongoing advancements in precision and adaptability. Quality control charts have undergone significant evolution from classical Shewhart charts^1^. The exponentially weighted moving average (EWMA) chart, devised by Roberts^2^, focuses on monitoring the process mean, while Page^3^ introduced the cumulative sum (CUSUM) chart to monitor process dispersion. In practical applications, monitoring changes in process dispersion holds more significance, as increased dispersion deteriorates the process, while decreased dispersion enhances process capability and productivity. Normalizing dispersion estimators through transformations aims to attain unbiased average run length (ARL) performance in control charts, crucial for effective out-of-control signal issuance. Unbiased ARL signifies that all out-of-control ARLs are lower than the in-control ARL. Shu and jiang^4^ introduce a novel EWMA dispersion chart (NEWMA) by truncating negative normalized observations to zero in the traditional EWMA statistic. Comparative analysis indicates that the NEWMA chart surpasses the traditional EWMA chart in detecting dispersion changes, particularly for small changes. Abbasi and Miller^5^ assesses the performance of eight control chart structures based on different process standard deviation estimates for monitoring process variability under normal and non-normal assumptions, offering guidance to quality practitioners. Castagliola et al.^6^ proposes precise bounds for double sampling S^2^ chart parameters with known process variance and explores the properties of the chart with estimated process variance. It compares average run length, standard deviation of run length, and average sample size, offering design guidelines and optimal procedures for both known and estimated process variance. Abujiya et al.^7^ focuses on EWMA charts using unbiased sample variance for monitoring upward shifts in process dispersion, employing simple random and ranked set sampling techniques. Monte Carlo simulations demonstrate superior performance compared to existing methods, with practical application illustrated using real industrial data. Saghir et al.^8^ proposed control chart, a generalization of existing charts, is evaluated for different sample sizes and smoothing constants, demonstrating superior performance in early detection of process variation shifts. The study compares the proposed modified EWMA chart with existing control charts, highlighting its efficiency, and provides a real-life dataset application. Haq^9^ proposed Max-AEWMA chart efficiently monitors mean and/or variance shifts in a normally distributed process, outperforming the Max-EWMA chart across various shift sizes. Comprehensive Monte Carlo simulations demonstrate the superior performance of the Max-AEWMA chart in terms of ARL SDRL. An illustrative example is provided for implementing both charts. Huwang et al.^10^ introduces two one-sided EWMA charts for detecting dispersion increases and decreases, along with a two-sided EWMA chart for simultaneous monitoring. Simulation studies reveal superior performance in detection sensitivity compared to existing counterparts for both increases and decreases in dispersion. Abbas et al.^11^ introduces two novel memory control charts, the floating T − S^2^ and floating U − S^2^, for monitoring process dispersion. Through simulation studies, these charts demonstrate superior ARL performance compared to CUSUM and EWMA charts for both positive and negative shifts. Arshad et al.^12^ of suggested a control chart using multiple dependent state (MDS) sampling for monitoring process variation, providing operational formulas for in-control and out-of-control ARLs. The proposed chart outperforms existing ones in timely detection of assignable causes, as demonstrated through ARL evaluations, and is applied to a real-life industrial example. Haq^13^ proposes an adaptive EWMA chart for monitoring dispersion shifts, based on an unbiased estimator and varying smoothing parameters. Through extensive Monte Carlo simulations, the AEWMA chart consistently outperforms existing competitors in detecting diverse shifts in process dispersion, demonstrating its superiority and providing practical insights through an illustrative example. The S^2^-GWMA control chart, employing a three-parameter logarithmic transformation, is proposed Alevizakosa et al.^14^ for monitoring process variability shifts. Monte Carlo simulations reveal its superior performance in detecting small to moderate upward shifts compared to existing charts, and a real example demonstrates its practical application. Chatterjee et al.^15^ S^2^-TEWMA control chart, incorporating a three-parameter logarithmic transformation of the sample variance, outperforms competing charts in detecting small shifts in process variability, as demonstrated through Monte Carlo simulations and illustrated in two practical examples. This paper presents a novel parameter free AEWMA control chart tailored for process dispersion monitoring. An exceptional characteristic involves estimating dispersion shifts through EWMA statistics and dynamically adjusting the smoothing constant based on the shift’s magnitude. Notably, the method used to determine this constant sets the AEWMA chart apart. Extensive Monte Carlo simulations played a pivotal role, providing crucial metrics like ARL and SDRL. These metrics facilitated a comprehensive assessment of the chart’s performance across diverse scenarios, ensuring a robust evaluation of its efficacy under varied conditions. This entailed a comparative examination with EWMA *S*^2^ and AEWMA *S*^2^ chart, with a particular focus on diminished ARL values. The proposed parameter free AEWMA-*S*^2^ concept showcases its superiority over the existing EWMA *S*^2^ and AEWMA *S*^2^ chart, particularly evident in practical industrial application using real-world data, extensively detailed in the example section. The paper unfolds as follows: section “Proposed parameter free AEWMA-S2 control chart” thoroughly develops the AEWMA-*S*^2^ chart, outlining its construction. Section “Discussion” rigorously assesses its performance, scrutinizing its effectiveness. Section “Performance comparison” incorporates the comparison of study. Section “Main findings” entails a main finding, illustrating examples discussed in section “Real-life application”. Finally, section “Conclusion remarks” encapsulates the study’s findings, drawing conclusive remarks on the research.

## Proposed parameter free AEWMA-S^2^ control chart

In this section, the AEWMA-*S*^2^ control chart is introduced to monitor variations in a process parameter *S*^2^. The variable *Y*, representing production outcomes, follows a normal distribution with a mean μ*Y* and variance: *Y* ∼ *N* (μ_*Y*_, $\sigma_{Y}^{2}$). Each *Yt* denotes the process outcome at a specific time *t*, forming a sequence {*Yt*} that tracks production outcomes over time. Initially, at *t*_0_, the process variance $\sigma_{Y}^{2}$, is in control, where {*Yt*} for *t* ≤ *t*_0_ conforms to *Yt* ∼ *N* (μ_*Y*_, $\sigma_{Y}^{2}$). However, when the process undergoes a shift denoted by δ, the variance changes to *Yt* ∼ *N* (μ_*Y*_, $\sigma_{1}^{2}$) for *t* > *t*_0_, where $\sigma_{1}^{2}$ indicates the altered process dispersion. The δ represents the ratio between the shifted process dispersion $\sigma_{1}^{2}$ and the original variance $\sigma_{Y}^{2}$, emphasizing the degree of change in $\sigma_{Y}^{2}$. A stable production process is characterized by δ = 1 for *t* ≤ *t*0, while δ ≠ 1 for *t* > *t*0 indicates a shift in the system’s behavior. This section explores the analysis of variations in production dispersion over time. A random sample of size *n* is extracted from the sequence {*Yt*} at a time *t* > *t*0, resulting in {*Y*1*t*, *Y*2*t*, …, *Ynt*}. Each *Yit* represents the *ith* observation within this sample. To investigate production variance, the process mean $\overline{{y_{t} }} = \sum\nolimits_{i = 1}^{n} {Y_{it} /n}$ and variance $S_{t}^{2} = \sum\nolimits_{i = 1}^{n} {(Y_{it} - \overline{Y}_{Yit} )^{2} /(n - 1)}$. To monitor the process variance, Castagliola^16^ suggested to apply the following transformation to $S_{t}^{2}$.

1 $$ T_{t} = a + b\, {\text{ln}}\left( {S_{t}^{2 } + c} \right). $$

here *a*, *b* and *c* > 0 are constants. The main vision of this approach is that if parameters *a*,*b* and *c* are selected in an arrangement that the transformation may consequence as the approximate normality to $T_{t}$ which is an improved approach than Hamilton and Crowder^17^. Castagliola^16^ demonstrated that the constants *a*,*b* and *c* are essentially equal to

2 $$ a = A\left( n \right) - 2B\left( n \right)\,ln\left( {\sigma_{o} } \right) $$

3 $$ b = B\left( n \right) $$

4 $$ c = C\left( n \right)\sigma_{o}^{2} $$

where only the sample size* n* is subjective on the three functions *A*, *B* and *C*. For more details, reader is refer to appendix 1 and 2 of Castagliola^18^. The approximation, denoted as ≈ *N* (0, 1), effectively generates an approximate standard normal distribution, simplifying the computation of unbiased ARL values. These unbiased ARL values play a pivotal role in overseeing the process of *Yt*, ensuring δ*t* = *E*(*Yt*) for the in-control process shift. Here, it is assumed that δ*t* = 0 at *t* ≤ *t*_0_, signifying an in-control state, while δ*t* ≠ 0 at *t* > *t*0 indicates an out-of-control system shifted state in σ*y*, signaling a modification in the system’s behavior. This approach ensures an impartial evaluation of ARL values, guaranteeing precise monitoring and detection of system shifts. After the normalization of values $S_{t}^{2}$, assessing the magnitude of the process dispersion δ poses a challenge since it is often unknown in practical scenarios. In the field of SPC, methodologies have been proposed to estimate this dispersion δ using unbiased estimators. For example, Jiang et al.^19^ recommended approximating or estimating the actual δ value, offering a practical approach to comprehend or evaluate the dispersion in the process.

5 $$ \delta_{t}^{*} = \psi T_{t} + \left( {1 - \psi } \right)\delta_{t - 1}^{*} , $$

The range of values utilized for the smoothing constant ψ to estimate the process δ extends from 0 to 1, denoted as ψ ∈ (0, 1]. Haq et al.^13^ utilized this as an impartial estimator of δ, aiming for

6 $$ \delta_{t}^{**} = \frac{{\delta_{t}^{*} }}{{1 - \left( {1 - \psi } \right)^{t} }} $$

with *E*($$\delta_{t}^{**}$$) = δ, aims to approximate or determine this δ value. According Haq et al.^13^, they extensively elaborate on these estimation techniques. When the process is in control (*E*($$\delta_{t}^{**}$$) = δ for *t* ≤ *t*_0_), indicating a phase without inherent dispersion δ, the estimation aligns with this zero value. However, in the event of a system shift where δ becomes relevant (*E* ($$\delta_{t}^{**}$$) = δ ≠ 0 for *t* > *t*_0_), the estimation identifies δ as positive ($$\delta_{t}^{**}$$ > 0) or negative ($$\delta_{t}^{**}$$ < 0) to denote increased or decreased δ magnitudes, respectively. For practical estimation, it is often recommended to use δ̃ *t* = $$\left| {\delta_{t}^{**} } \right|$$. This approach ensures consistent estimation, regardless of whether the dispersion δ magnitude increases or decreases, providing a standardized representation for assessing process dispersion.

The suggested AEWMA-S^2^ statistic using the sequence $\{ Y_{t} \}$ for monitoring process variance is given by

7 $$ F_{t} = g\left( {\hat{\delta }_{t} } \right)T_{t} + \left( {1 - g\left( {\hat{\delta }_{t} } \right)} \right)F_{t - 1} $$

where $g\left( {\hat{\delta }_{t} } \right) \in \left( {0,\left. 1 \right]} \right.$ and $F_{0} = 0$ such that

8 $$ \left( {\hat{\delta }_{t} } \right) = \left\{ {\begin{array}{*{20}l} {\frac{1}{{a\left[ {1 + \left( {\hat{\delta }_{t} } \right)^{ - c} } \right]}}} \hfill & {\quad if\quad 0 < \hat{\delta }_{t} \le 2.7} \hfill \\ 1 \hfill & {\quad if\quad \hat{\delta }_{t} > 2.7} \hfill \\ \end{array} } \right.. $$

Sarwar et al.^20^ introduced the function given in (5) to adapt the value of smoothing constant based on the estimated shift. The constants used in $g(\hat{\delta }_{t} )$ are suggested as $a = 7$ and $c = 1$, when $1 < \hat{\delta }_{t} \le 2.7$, the value of $c = 2$ for $\hat{\delta }_{t} \le 1$. The process is said to be out-of-control if the plotting statistic of exceeds the threshold value $h$; otherwise, the process is in control.

If the recommended statistic F_*t*_ exceeds *h* or falls below *h* in a one-sided AEWMA-*S*^2^ chart, crossing the assumed positive decision interval *h* (*h* > 0), it triggers an out-of-control status for the process. Conversely, if it does not breach these thresholds, the process remains in control. The threshold h serves as a specific limit for a given *n*, and ψ’s value is chosen to ensure the in-control ARL guarantees optimal sensitivity for the proposed chart statistic Ft at a predefined fixed *ARL*0 level. For each set of *n* and ψ, a distinct h value is calculated. Determining whether the monitoring system should detect an increased or decreased pattern relies on the insights gained during phase I. This approach ultimately minimizes sampling costs and human effort in the monitoring process. The combination of parameters ψ and *n* significantly influences the optimized threshold performance for specified in-control run-lengths, as highlighted by Haq^12^, considering a predefined δ. The h value serves to establish the in-control ARL as *ARL*0, employing an adaptive function-based approach as recommended. Employing smaller ψ values aims to efficiently detect smaller magnitudes of δ while maintaining the capability to detect larger process δ, as elaborated in the subsequent section.

## Discussion

The evaluation of a control chart’s performance involves assessing its reliability attributes, including mean, standard deviation, and percentiles of the run length (RL). Various techniques, such as the Probability method, Markov chain, Integral equations, and Monte Carlo (MC) simulation, can be utilized to determine these attributes. In this context, we specifically employed the MC simulation method, which is widely acknowledged and utilized for calculating RL profiles in the proposed AEWMA control chart. To obtain RL profiles, we conducted sampling from a normal distribution with a specified mean (δ) and variance of 1. The values of δ were systematically varied, covering a range of scenarios, with ψ values set at 0.15 and 0.20. This comprehensive approach allowed us to assess and understand the performance of the AEWMA control chart under different conditions, ensuring a thorough analysis of its reliability characteristics.

The analysis reveals that the performance of the proposed parameter free AEWMA control chart is superior when considering zero-state reliability profiles, including ARL, SDRL, and percentiles. This enhanced performance is particularly evident as the parameter δ, representing shifts in the process dispersion, varies. Comprehensive details and specific values can be found in Tables 1, 2, 3, and 4, providing a comprehensive understanding of the AEWMA control chart’s behavior under different conditions. Furthermore, a concise discussion is presented to offer insights into the overall findings and implications of the study.

- Tables 1, 2, 3, and 4 provide a comprehensive analysis of the AEWMA-S^2^ control chart’s ARL and SDRL at fixed ψ values. The results reveal a clear trend: as the value of δ increases, both ARL and SDRL tend to decrease, and conversely, as δ decreases, ARL and SDRL show an increasing pattern. For instance, in Table 1, with ψ fixed and varying values of δ (0.25, 0.80, 1.10, 1.40, 2.00), the corresponding ARL1 values are 6.26, 36.04, 42.03, 7.32, and 2.83, while SDRL1 values are 0.44, 13.28, 36.09, 4.12, and 1.30, respectively, at ARL_0_ = 370. This trend is consistent across different δ values and is similarly observed at ARL_0_ = 500, highlighting the inverse relationship between δ, ARL, and SDRL in the context of the AEWMA-S^2^ control chart.
- Tables 1, 2, 3, and 4 reveal a consistent inverse relationship between RL percentiles and the parameter δ in the context of the AEWMA control chart. For instance, examining Table 2, with an In-Control (IC) ARL set at 500, varying values of δ (0.50, 0.80, 1.10, 2.00) showcase the following percentiles (P_05_, P_10_, P_25_, P_50_, P_75_, P_90_, P_95_): (9, 9, 10, 11, 13, 14, 15), (21, 23, 29, 36, 46, 58, 66), (8, 11, 19, 36, 64, 101, 130), and (1, 2, 2, 3, 4, 5, 6) respectively. This consistent pattern is observed in Tables 1, 3, and 4, emphasizing the influence of δ on RL percentiles in the AEWMA control chart.
- The value of the “*h”* and IC ARL is directly related, as expected and the r codes are included in Appendix A.

**Table 1:** Run-length profile of the offered parameter free AEWMA-S^2^ chart applying two-sided for *ARL*_0_ = 370, with *n* = 5.

Shift	ARL	SDRL	P_05_	P_10_	P_25_	P_50_	P_75_	P_90_	P_95_
0.25	6.26	0.44	6.0	6.0	6.0	6.0	7.0	7.0	7.0
0.50	10.79	1.81	8.0	9.0	10.0	11.0	12.0	13.0	14.0
0.75	26.94	8.19	16.0	18.0	21.0	26.0	32.0	38.0	42.0
0.80	36.04	13.28	19.0	22.0	27.0	34.0	43.0	53.0	61.0
0.85	54.28	26.03	24.0	28.0	36.0	48.0	66.0	88.0	105.0
0.90	104.13	68.64	33.0	40.0	56.0	85.0	131.0	193.0	238.0
0.95	313.31	277.10	43.0	63.0	117.0	228.0	422.0	675.0	862.0
1.00	370.28	375.78	20.0	37.0	103.0	257.0	515.0	848.0	1114.0
1.10	42.03	36.09	7.0	10.0	17.0	31.0	56.0	89.0	114.0
1.20	17.04	11.82	4.0	6.0	9.0	14.0	22.0	33.0	40.0
1.30	10.26	6.24	3.0	4.0	6.0	9.0	13.0	18.0	22.0
1.40	7.32	4.12	2.0	3.0	4.0	6.0	9.0	13.0	15.0
1.50	5.70	3.04	2.0	3.0	4.0	5.0	7.0	10.0	12.0
1.60	4.72	2.37	2.0	2.0	3.0	4.0	6.0	8.0	9.0
1.70	3.99	1.95	2.0	2.0	3.0	4.0	5.0	7.0	8.0
1.80	3.51	1.68	1.0	2.0	3.0	4.0	5.0	6.0	7.0
2.00	2.83	1.30	1.0	1.0	2.0	3.0	3.0	5.0	5.0
2.50	2.00	0.88	1.0	1.0	1.0	2.0	2.0	3.0	4.0

**Table 2:** Run-length profile of the offered parameter free AEWMA-S^2^ chart applying two-sided for *ARL*_0_ = 500, with *n* = 5.

Shift	ARL	SDRL	P_05_	P_10_	P_25_	P_50_	P_75_	P_90_	P_95_
0.25	6.63	0.50	6.0	6.0	6.0	7.0	7.0	7.0	7.0
0.50	11.39	1.89	9.0	9.0	10.0	11.0	13.0	14.0	15.0
0.75	28.72	8.69	17.0	19.0	23.0	28.0	33.0	40.0	45.0
0.80	38.79	14.42	21.0	23.0	29.0	36.0	46.0	58.0	66.0
0.85	59.90	29.03	26.0	30.0	39.0	53.0	73.0	97.0	115.0
0.90	121.57	82.99	36.0	44.0	64.0	98.0	155.0	229.0	288.0
0.95	406.83	367.50	54.0	78.0	148.0	294.0	549.0	884.0	1139.0
1.00	500.35	506.19	26.00	49.00	138.00	344.00	701.00	1163.00	1513.05
1.10	48.27	41.25	8.0	11.0	19.0	36.0	64.0	101.0	130.0
1.20	18.82	12.74	5.0	6.0	10.0	16.0	24.0	35.0	43.0
1.30	11.25	6.63	4.0	4.0	7.0	10.0	14.0	20.0	24.0
1.40	7.98	4.37	3.0	3.0	7.0	8.0	10.0	14.0	16.0
1.50	6.19	3.17	2.0	3.0	4.0	6.0	8.0	10.0	12.0
1.60	5.06	2.52	2.0	2.0	3.0	5.0	6.0	8.0	10.0
1.70	4.29	2.04	2.0	2.0	3.0	4.0	5.0	7.0	8.0
1.80	3.77	1.76	2.0	2.0	2.0	3.0	5.0	6.0	7.0
2.00	3.04	1.35	1.0	2.0	2.0	3.0	4.0	5.0	6.0
2.50	2.14	0.91	1.0	1.0	2.0	2.0	2.0	3.0	4.0

**Table 3:** Run length results of suggested parameter free AEWMA-S^2^ chart under one-sided for monitoring increase in dispersion at *ARL*_0_ = 370.

Shift	ARL	SDRL	P_05_	P_10_	P_25_	P_50_	P_75_	P_90_	P_95_
1.00	370.56	385.95	16.0	30.0	91.0	245.0	513.0	872.1	1155.0
1.05	86.62	85.29	9.0	13.0	26.0	59.0	118.0	199.0	257.0
1.10	38.14	33.42	6.0	8.0	15.0	28.0	51.0	81.0	106.0
1.20	15.75	11.02	4.0	5.0	8.0	13.0	21.0	30.0	37.0
1.30	9.65	5.91	3.0	4.0	5.0	8.0	12.0	17.0	21.0
1.40	6.86	3.90	2.0	3.0	4.0	6.0	9.0	12.0	14.0
1.50	5.40	2.97	2.0	2.0	3.0	5.0	7.0	9.0	11.0
1.60	4.42	2.30	2.0	2.0	3.0	4.0	6.0	8.0	9.0
1.70	3.78	1.91	2.0	2.0	2.0	3.0	5.0	6.0	7.0
1.80	3.33	1.64	1.0	2.0	2.0	3.0	4.0	5.0	6.0
2.00	2.68	1.27	1.0	1.0	2.0	2.0	3.0	4.0	5.0
2.50	1.90	0.86	1.0	1.0	1.0	2.0	2.0	3.0	3.0
3.00	1.53	0.65	1.0	1.0	1.0	1.0	2.0	2.0	3.0

**Table 4:** Run length results of suggested parameter free AEWMA-S^2^ chart under one-sided for monitoring increase in dispersion at *ARL*_0_ = 500.

Shift	ARL	SDRL	P_05_	P_10_	P_25_	P_50_	P_75_	P_90_	P_95_
1.00	500.97	511.16	22.0	43.0	129.0	337.0	697.0	1177.0	1524.0
1.05	105.77	100.88	11.0	17.0	33.0	74.0	146.0	238.0	306.0
1.10	44.76	37.89	8.0	11.0	18.0	33.0	60.0	95.0	120.0
1.20	17.62	12.08	4.0	6.0	9.0	14.0	23.0	33.0	41.0
1.30	10.61	6.38	3.0	4.0	6.0	9.0	14.0	19.0	23.0
1.40	7.57	4.18	3.0	3.0	5.0	7.0	10.0	13.0	16.0
1.50	5.90	3.10	2.0	3.0	4.0	5.0	7.0	10.0	12
1.60	4.85	2.42	2.0	2.0	3.0	4.0	6.0	8.0	9.0
1.70	4.10	1.99	2.0	2.0	3.0	4.0	5.0	7.0	8.0
1.80	3.61	1.69	2.0	2.0	2.0	3.0	4.0	6.0	7.0
2.00	2.92	1.32	1.0	2.0	2.0	3.0	4.0	5.0	5.0
2.50	2.06	0.89	1.0	1.0	1.0	2.0	3.0	3.0	4.0
3.00	1.64	0.71	1.0	1.0	1.0	2.0	2.0	3.0	3.0

## Performance comparison

In the standard evaluation of a control chart, its statistical presentation is typically assessed through its RL profiles, encompassing mean, standard deviation, and percentiles of RLs. A control chart is considered superior if its out-of-control ARL is smaller than that of other charts, given a specific in-control ARL and the magnitude of the shift. This study compares the proposed parameter-free AEWMA-S^2^ control chart with EWMA-S^2^ and AEWMA-S^2^ control charts in terms of RL profiles, including zero-state, under various dispersion shift sizes. In the field of SPC, it is well-established that adaptive charts tend to be more sensitive than non-adaptive ones in detecting shifts within a specified range, thus offering enhanced protection. Haq et al.^13^ introduced an AEWMA-S^2^ control chart wherein they estimated the shift using a bias-free estimator. Subsequently, the value of the smoothing constant for plotting the EWMA statistic was selected through a step function. They claimed that their proposed control chart outperforms existing AEWMA, Cumulative Sum (CUSUM), Adaptive CUSUM (ACUSUM), and Double CUSUM (DCUSUM) charts. As part of this comparison, the proposed control chart is evaluated against the AEWMA control chart. The RL profiles of AEWMA-S^2^ and the proposed parameter-free AEWMA-S^2^ control charts are determined using Monte Carlo simulations with 100,000 iterations. The in-control RL is set at ARL_0_ = 370 and 500 for comprehensive analysis and comparison.

Table 5 illustrates the superior performance of the proposed AEWMA-S^2^ chart compared to the EWMA-S^2^ and AEWMA-S^2^ charts at various shift values with an in-control ARL_0_ set at 370. For instance, the proposed AEWMA-S^2^ chart, with δ values of 1.10 and 1.06, yields ARL values of 253.73, 71.32, and SDRL values of 258.69, 68.09. In contrast, the EWMA-S^2^ control chart provides ARL values of 303.0, 135.66, and SDRL values of 291.85, 131.55. Similarly, for AEWMA-S^2^ control chart, the run length values are ARL = 270.11, 90.88, and SDRL = 304.01, 94.06. The trend continues at various shift magnitudes (e.g., δ = 1.50 and 2.00), where the proposed AEWMA-S^2^ control chart consistently outperforms its counterparts, as demonstrated in the provided data.

**Table 5:** ARL and SDRL outcomes for comparative analysis in process dispersion.

Shift	EWMA-S^2^ *n* = 5		Haq et al.^13^ AEWMA-S^2^, *n* = 5		Proposed parameter free AEWMA-S^2^ *n* = 5	
	ARL	SDRL	ARL	SDRL	ARL	SDRL
1.00	370.99	362.97	370.89	424.91	370.56	385.95
1.01	303.00	291.85	270.11	304.01	253.73	258.69
1.02	254.21	254.47	204.87	225.40	188.45	197.64
1.03	215.34	208.96	158.46	172.71	142.19	143.12
1.04	182.94	176.77	128.36	136.44	110.90	111.50
1.05	159.36	156.89	108.09	113.00	86.62	85.29
1.06	135.66	131.55	90.88	94.06	71.32	68.69
1.07	120.79	116.08	77.01	79.47	59.08	54.59
1.08	104.83	99.73	67.13	68.21	50.24	50.24
1.50	9.81	6.54	6.47	5.92	5.90	3.10
2.00	2.92	1.30	2.84	2.81	2.72	1.32

## Main findings

Based on the analysis of Tables, key observations regarding the proposed parameter-free AEWMA-S^2^ control chart can be emphasized as follows:

1. The proposed control chart is well-suited for the rapid detection of minor shifts in the dispersion of a production process, particularly when the variable of interest adheres to a normal distribution. For instance, as depicted in Table 5, when a shift of 1.10 occurs, the ARL of the proposed chart is notably lower at 253.73. In comparison, the ARL values for the existing EWMA and AEWMA control charts are higher at 303.00 and 270.11, respectively. This substantiates the efficacy of the proposed chart in swiftly identifying subtle shifts in process dispersion.
2. Upon examination of Tables 1, 2, 3, and 4, it is evident that the ARL values exhibit a decreasing trend with an increase in the dispersion shift. This pattern indicates that the chart efficiently detects process shifts early on, especially with larger changes in dispersion. For instance, referring to Table 4, at a shift of 1.20, the ARL is 17.62, while at a mean shift of 1.60 under the same conditions, the ARL further decreases to 4.85. This underscores the effectiveness of the chart in promptly identifying both minimal and substantial shifts in dispersion.
3. Upon detailed analysis of the tables, it is evident that the proposed parameter-free AEWMA-S^2^ control chart outperforms its counterparts in terms of smaller RL profiles, encompassing ARL, SDRL, and percentiles. This superior performance is notable when the shift, regardless of its magnitude, affects the process dispersion. Additionally, the proposed chart effectively addresses the challenge of a high false alarm rate during zero-state conditions.

## Real-life application

In this segment, we presented the real-world implementation of the proposed AEWMA-S^2^ control chart for monitoring dispersion. A genuine dataset sourced from Montgomery^21^ is employed, specifically concentrating on the flow width of wafers measured in microns during the hard bake phase and photolithography stages of semiconductor manufacturing. Phase I involves the meticulous collection of 25 samples, each containing 5 measurements at hourly intervals. The primary statistical parameter derived from these samples is the variance, offering valuable insights into the variability within each set of measurements.

**Figure 1:**
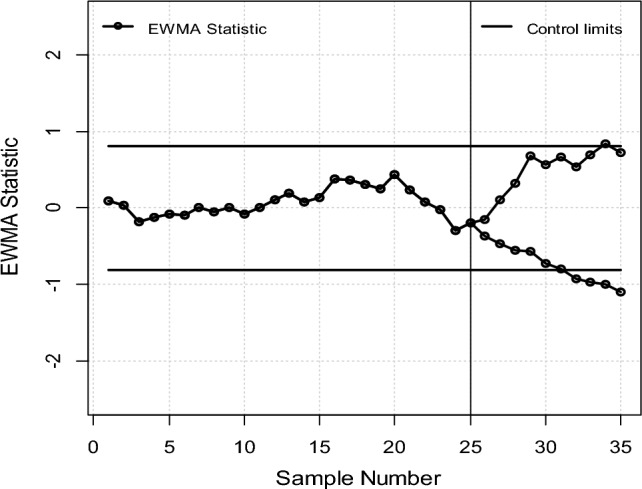
Plot for the suggested EWMA-S^2^ control chart.

**Figure 2:**
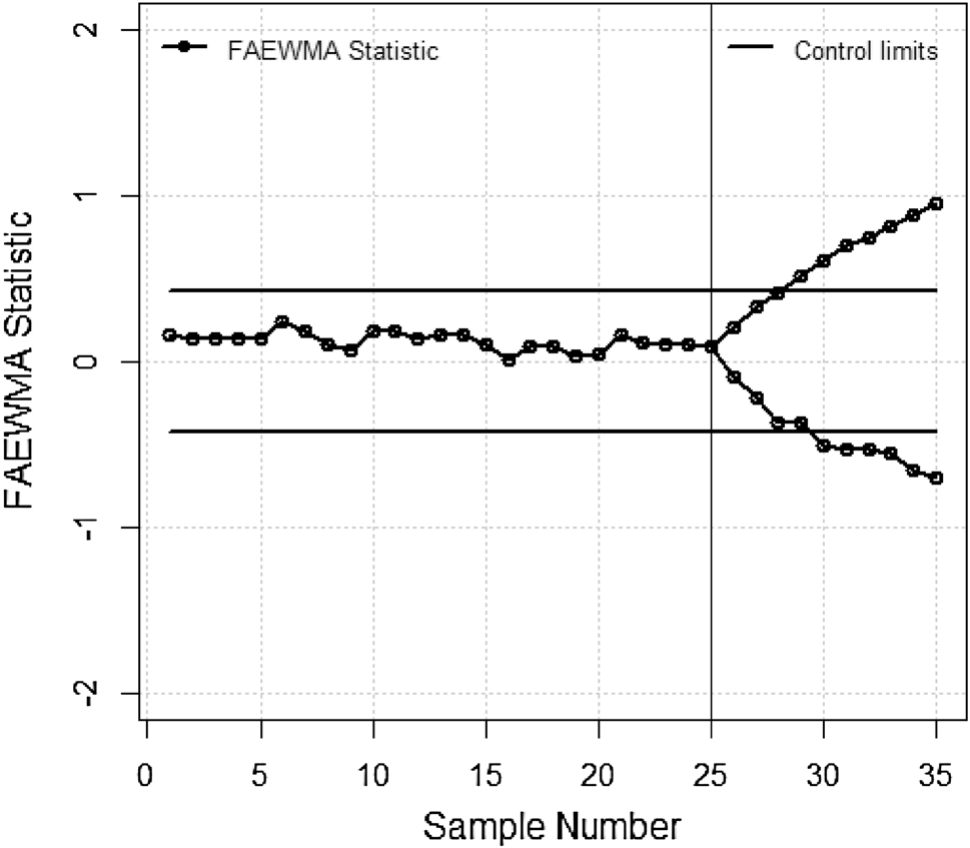
Plot for the proposed AEWMA-S^2^ control chart.

The initial 25 samples are considered in-control with *t*_0_ = 25, while the subsequent 10 samples form a phase II shifted dataset with *t*_1_ = 10, deliberately subjected to a dispersion δ to showcase the swift detection capability of the proposed statistic. Consequently, sample means and variances are computed as follows: $\overline{{y_{t} }} = \sum\nolimits_{i = 1}^{n} {\overline{Y}_{Yit} /n}$ and $S_{t}^{2} = \sum\nolimits_{i = 1}^{n} {(Y_{it} - \overline{Y}_{Yit} )/(n - 1)}$ for *t* = 1, 2, …, 25 and *i* = 1, 2, …, 5, where *Yit* denotes the ith observation in the tth sample of phase I. Using the phase I data, the overall process mean μ is estimated as $\overline{Y} = \sum\nolimits_{i = 1}^{{t_{0} }} {\overline{Y}_{t} /t_{0} }$ and the process variance σ^2^ as $\overline{S}_{t}^{2} = \sum\nolimits_{i = 1}^{{t_{0} }} {S_{t}^{2} /t_{0} }$ are also calculated. For the phase II shifted dataset, data is adjusted as *Y* ∗ $it = Y + {\varvec{\delta}}(\overline{Y}\,it - \overline{Y})$ for δ values of 0.25 and 1.75 across all observations. The shift in the plotted statistics for the proposed chart in Fig. 1 illustrates fluctuations in the process, capturing both upward and downward trends. Figure 1 indicates that during the initial 25 samples both charts remained within control affirming production stability. However, after the 25th observation with the remaining shifted 10 samples the proposed charts promptly reflected the impact of δ on process dispersion displaying immediate upward and downward trends. Notably, Fig. 1 show exiting EWMA-S^2^ dispersion chart identify increasing and decreasing δ at the 34th and 31th points, respectively. While the offered parameter free AEWMA-S^2^ dispersion chart detected increasing and decreasing δ at Fig. 2 detect out-of-control on 28th and 30th points respectively. This highlights the effectiveness of the proposed AEWMA-S^2^ dispersion chart in swiftly identifying dispersion δ, indicating its potential for practical implementation across various industries.

## Conclusion remarks

The realm of adaptive control charts has gained significant prominence due to their heightened sensitivity in promptly identifying deviations in the process (denoted by δ). These adaptive charts exhibit superior sensitivity compared to conventional Shewhart and EWMA mean and dispersion monitoring charts, particularly for small and moderate δ values. They are intricately designed to swiftly detect process δ within specified ranges, proving especially advantageous for industries where detecting even minor deviations is crucial. In industries like pharmaceuticals, automotive, food production, packaging, and automation, where small variations in δ can have serious implications, the introduction of a novel adaptive EWMA-S^2^ dispersion chart, termed parameter-free AEWMA-S2, becomes highly relevant. The objective of this chart is to efficiently and promptly monitor frequent shifts of any magnitude within specified δ intervals, outperforming the current AEWMA-S^2^ dispersion chart. The efficacy of the proposed AEWMA-S^2^ chart is substantiated by its shorter run-length profiles, extensively assessed through Monte Carlo simulations in R software and presented in tabular format. Upon examination of the outcomes, the suggested chart consistently exhibits superior performance in comparison to the existing AEWMA chart across diverse δ magnitudes, showcasing efficiency in scenarios involving small, moderate, and large δ variations. As a suggestion, it can be proposed for more effective surveillance of alterations in process dispersion compared to established approaches. Extending this method to a Bayesian framework with the normal distribution could substantially broaden its applicability in various domains of medical sciences. Its versatility in diverse scenarios, including clinical trials, disease prognosis, or assessing treatment effectiveness, could provide valuable insights, assisting in decision-making processes and fostering a deeper comprehension of patterns in medical data.

### Supplementary Information

Supplementary Information.

## Data Availability

The corresponding author possesses the datasets employed or scrutinized in the recent study and can provide access to interested parties upon a reasonable request. This ensures that individuals seeking the data for further analysis or validation can contact the corresponding author to obtain the requisite information.
