# Supplementary material for: Parameter free AEWMA control chart for dispersion in semiconductor manufacturing
Source: Sci Rep. 2024 May 7;14:10512. doi: 10.1038/s41598-024-61408-5 (PMC11076580; doi:10.1038/s41598-024-61408-5)
Supplement: Supplementary file 1 — Supplementary Information. [file 41598_2024_61408_MOESM1_ESM.docx]

**Appendix A**

#correct version of ARL, SDRL and percentiles.

rm(list=ls())

library(MASS)

zh=c();zp=c();AEWMA=c();

Z0=0;rl=c();sig=1;T=c();p=1.08;b=0.0;mu=0+b*sig;

Mx=c(); ucl=c(); rl=c();zp1=c();muM=muy=0; sigyy=1;y11=list();x1=c();x2=c();

ld1=0.10;ld2=0.10; h =0.5547;zt=c();Sx=c();R=c();w1 = 3.71902;

w2 = 4.09023; w3 = 5.09023;

n=5; a=-0.8969; b=2.3647; c=0.5979;

Z0=a+b*log(1+c,exp(1))

for(j in 1:20000)

{

for(i in 1:10000)

{

x=rnorm(n,mu,sig*p)

T=a+b*log(var(x)+c,exp(1))

zp1[i]=abs(T)

if(zp1[i] >5.1) {sci=1}

else

if(zp1[i] >0 && zp1[i] <= 5.1) {sci=1/(10*(1+(zp1[i])^-2))}

else {sci=0}

if (i==1)

{AEWMA[i] =sci*T+(1-sci)*Z0;}

else{AEWMA[i] =sci*T+(1-sci)*AEWMA[i-1];}

#if(abs(AEWMA[i]) > h)

if((AEWMA[i]) > h)

#if((AEWMA[i]) < h)

{rl[j]=i;break;}

else{rl[j]=0;}

}

}

mean(rl)

sd(rl)

quantile(rl, c(0.05, 0.10, 0.25, 0.50,0.75,0.90,0.95))
